# Iconographic Professional Interests Inventory (3IP): A New Validation Study

**DOI:** 10.5964/ejop.v11i4.927

**Published:** 2015-11-27

**Authors:** Diego Boerchi, Paola Magnano

**Affiliations:** a Faculty of Education, Department of Psychology, Università Cattolica del Sacro Cuore, Milano, Italy; b Faculty of Human and Social Sciences, Università Kore, Enna, Italy; Academy of Special Education, Warsaw, Poland

**Keywords:** vocational interests development, vocational interests assessment, children, Holland’s model, psychometrics

## Abstract

Interests have been a central focus of counselling psychology (and vocational psychology in particular) for over 100 years. The awareness of professional interests increases self-knowledge and provides occupational information. In career counselling, vocational interests are assessed more frequently than any other vocational construct, though early evaluations (before 13 years old) of professional interests are very rare. The aim of this research is to examine the 3IP construct (Iconographic Professional Interests Inventory; an inventory composed of 65 stylised pictures that represent people in the act of performing a job) in depth, testing more models in addition to the 19 vocational areas proposed in the 3IP manual. Results show that most of the vocational areas can be grouped into 4 second-level areas (“things”, “people”, “leisure”, and “culture”). Moreover, Holland’s RIASEC model is tested; an accurate selection of items reveals that this model works well using 24 specific jobs. The research concludes that the inventory has good psychometric qualities which can grow further by mean of the increasing, in a targeted way, of the number of jobs.

It is widely assumed that vocational interests play an important role in satisfaction and achievement, both in study and work. Vocational guidance has historically considered both aptitude and interests as the fundamental psychological dimensions to consider in the training or job choice process. Both research and vocational interventions usually concentrate on the first choice of a study, which does not take place with students younger than 13 years old. Research into interest development is rare, but it is particularly rarely studied among children; this is in part due to the lack of assessment instruments developed to work with this target.

The aim of this research is to deepen knowledge of a new instrument developed to appositely investigate professional interests for children.

## Definitions

*Dispositional interests*, as opposed to *situational interests* (Silvia, 2006), are trait-like, reflecting a person’s preferences for behaviours, situations, contexts in which activities occur, and/or the outcomes associated with these preferred activities (Rounds, 1995).

From a dispositional perspective, we can identify *academic* and *vocational* interests (Savickas, 1999). Academic interests are associated with facilitative effects on cognitive functioning, learning, and academic achievement (Schiefele, Krapp, & Winteler, 1992). The educational psychology tradition of interest research focuses on the relationship between interest and attention/motivation, primarily examining interest’s function in learning and academic achievement for children’s classroom settings (Krapp, 1999; Renninger, Hidi, & Krapp, 1992). Holland (1966) defined vocational interests as “the expression of personality in work, hobbies, recreational activities, and preferences” (p. 3). School-subject preferences and choices are systematically related to vocational interests (Elsworth, Harvey-Beavis, Ainley, & Fabris, 1999). Holland (1966) proposed in his person-environment fit theory of occupational choice that vocational choices arise from perceptions of fit between work environment and interests; vocational interests facilitate a fit between people and their environment to improve occupational success and satisfaction. Hereafter, “vocational interest” will be used to refer both to academic and vocational interests.

## Vocational Interests Development

Despite their dispositional qualities, vocational interests develop like other personal factors (Roe, 1956; Tyler, 1961). Evidence indicates that vocational interests change in response to the positive and negative environmental reinforcements they receive, especially the evaluations of significant others (e.g., parents, teachers, other caregivers, and peers), reinforcements, one’s own attributions, and social and cultural forces. Other powerful influences include gender (Betz & Schifano, 2000; Boerchi, 2011; Eccles, 1993), racial/ethnical groups (Leong, 1995), and levels of socioeconomic status (Bandura, Barbaranelli, Caprara, & Pastorelli, 2001). Macro-level factors, such as economics and public policy, also contribute to the development of interests (Blustein, Phillips, Jobin-Davis, Finkelberg, & Roarke, 1997).

There is general consensus that vocational interests emerge during childhood (Tracey, 2001) and develop by an increasingly refined differentiation of activities and preferences (Tyler, 1964). Interest differentiation does not occur at a single time or at a particular level; rather, it seems to be a continuous process that follows a set of steps. The differentiation process is likely affected by different kinds of childhood experiences (Roe, 1957). It probably stops at a higher general level for nonpreferred areas but continues to lower and more detailed levels for the preferred areas.

The notion that adolescents become more certain of their interests with age has received empirical support (e.g., Cook et al., 1996; Csikszentmihalyi & Schneider, 2000) and has been integrated into career-development theories (e.g., Ginzberg, Ginsburg, Axelrad, & Herma, 1951; Gottfredson, 1981, 2002; Holland, 1997; Super, Savickas, & Super, 1996; Todt & Schreiber, 1998).

### In Childhood

Children are exposed to occupational images at an early age and develop relatively steady career aspirations as early as kindergarten (McMahon & Patton, 1997). From a very young age, children are inundated with cultural imagery of occupations through the media and other sources (Wright et al., 1995). From the time children are able to speak, their interests are frequently elicited through inquires about their aspirations (e.g., “What do you want to be when you grow up?”; Trice & Rush, 1995). According to Hartung, Porfeli, and Vondracek (2005), a significant amount of literature has examined aspects of children’s vocational development and behaviour since the mid-twentieth century, when theorists such as Ginzberg et al. (1951), Roe (1956), and Super (1957) explicitly conceptualised childhood as an important formative period for vocational development:

As childhood ideas about work and occupations are the precursors to adolescent career development and later exploration of the world of work, it behoves us to continue to expand our understanding of career development during the elementary and middle school years. (Howard & Walsh, 2011, p. 257)

### In Adolescence

It is generally held that interests are quite stable over time (Hansen, 1984; Swanson, 1999) and, furthermore, that interests become more fully developed and relatively stable by late adolescence (Hansen, 1984; Tracey, 2002a; Tracey, Robbins, & Hofsess, 2005). Early adolescence, from 12 to 14 years old (middle school), is particularly significant for changes in the cognitive and social fields across all ethnic groups and both genders (Nichols, 1990; Phillips & Zimmerman, 1990; Ruble & Seidman, 1996). Children are in the process of moving from a more concrete representation of interests to a more abstract representation that is commonly utilised by adults (Tracey, 2002a; Tracey & Ward, 1998). With age, there is increasing realism in occupational choice (Osipow, 1983) and in perceptions of competence (Nichols, 1990; Phillips & Zimmerman, 1990). Marsh and Yeung (1998) have posited that these changes are the result of the importance that internal and external referents assume as age increases. In the middle school the social comparison is an important way to build self-perception, so children frequently notice how others are respect to interests and competencies. Then, this externally driven focus is used by children to evaluate their own interests and skills to each other, in an ipsative way. This process contributes to modify the structure and the level of interests and competency rating at this age (Tracey, 2002a). These changes also have an important consequence on how students see themselves: their self-evaluations become increasingly differentiated, critical, and realistic with age (Harter, 1990; Marsh, 1987; Nichols, 1990). Indeed, children in the middle school ages (e.g., 12-year-olds) start to focus on competencies in particular domains; they discriminate by area and by comparing themselves with other children (Harter, 1990). Afterwards, interests tend to crystallise, and by high school, interests assume a structure that is invariant across older ages (Tracey & Rounds, 1993).

### RIASEC Model

Holland (1966, 1997) proposed a model of vocational interests that specifies six interest domains: realistic (R), investigative (I), artistic (A), social (S), enterprising (E), and conventional (C), hereafter referred to as RIASEC. The RIASEC model represents the six interest domains in a two-dimensional space by arranging them in a hexagon in which similarity between interests determines their position and proximity. Interest domains are located next to those to which they are most similar and opposite those to which they are most dissimilar. As Nagy, Trautwein, and Lüdtke (2010) highlighted, the hexagonal model has stimulated a wealth of research. Early reviews supported Holland’s model (Prediger, 1982; Rounds, 1995), and quantitative investigations have largely confirmed these findings (e.g., Day, Rounds, & Swaney, 1998). In particular, a structural meta-analysis conducted by Tracey and Rounds (1993) confirmed that the circumplex hexagonal model has a good fit in U.S. samples. In contrast to the relatively clear pattern of findings from the United States, findings from other countries have not been definitive. Most early qualitative reviews concluded that cross-cultural generalisations of the circular model are valid (Hansen, 1987; Harrington & O’Shea, 1993; Holland, 1985). However, the majority of quantitative investigations conducted outside the United States have failed to confirm Holland’s model (e.g., Fouad & Dancer, 1992; Glidden-Tracey & Parraga, 1996; Swanson, 1992). In addition, Šverko and Babarović (2006) concluded that, in almost all non-U.S. countries and even in the U.S. ethnic sample, the structure of vocational types could not be described with the circular model. The model’s degree of fit has been shown to differ a great deal from country to country (Einarsdóttir, Rounds, Ægisdóttir, & Gerstein, 2002; Farh, Leong, & Law, 1998; Leong, Austin, Sekaran, & Komarraju, 1998; Rounds & Tracey, 1996; Tracey & Rounds, 1993). Moreover, RIASEC’s circular structure is less well-established in international contexts and in children. In fact, Tracey and Ward (1998) found that the circular structure is less prevalent in children under 14 years; that study demonstrated that the circular structure does not hold well for younger children, though adherence to the normative circular model increases with age. The circular structure of interests seems to develop during middle school and remain stable in high school (see also Tracey, 2002b; Tracey, Lent, Brown, Soresi, & Nota, 2006; Tracey & Robbins, 2005). Thus, the circular structure varies depending on both culture and developmental level, suggesting that Holland-based scales may not have a common meaning across all cultures or age levels.

### CCCA Model

Howard and Walsh (2010) proposed the conceptions of career choice and attainment (CCCA) model to study and explain the development of children’s reasoning about careers. The CCCA model, which was derived from theory and research on children’s reasoning related to illness, violence, and ethnic identity, uses three approaches to cognitive reasoning.

In the first approach, *association*, children use fantasy to think about careers. They engage in little self-reflection and have too little self-awareness to gather information about preferences, abilities, and opportunities. Future career choices are based on heroes or imaginary models. The association approach is divided into two levels, *pure association* and *magical connection*, and it is typical of kindergarten students (5–6 years old).

In the second approach, *sequence*, children are able to identify an agent (e.g., activity, event, situation, or condition) that leads to choosing and attaining a job. Career choice and attainment are understood as separate processes, and children are able to explain how the two are related in concrete terms, using direct spatial contact or temporal sequences (e.g., get a diploma, go to college, and then start the job). The sequence approach is divided into two levels, *external activities* and *internal processes and capacities*, and it is typical of 3rd grade students (8–9 years old).

In the third approach, *interaction*, children are able to define the act of choosing a career as a process that involves dynamic interaction between self-awareness of personal attributes (e.g., interests, innate abilities, skills, and values) and environmental opportunities (e.g., availability of a career, opportunities to develop the requisite skills, and the current conditions of the job market). Attaining a career is considered to be a process based on the dynamic interaction of personal characteristics and career characteristics. The interaction approach is also divided into two levels, *interaction* and *systemic interaction*, and it is typical of 6th grade students (11–12 years old).

### The Role of Gender in Vocational Interests Development

Holland suggested that interests are also determined by gender. This concept is supported by much theory and research which considers gender to be a key factor in vocational development (Stockard & McGee, 1990). According to Havighurst (1983), children acquire a gender-role identity via family, peer groups, and school experiences. These experiences may also encourage gender-role stereotyping (Birk & Blimline, 1984; Feingold, 1988; McKenna & Ferrero, 1991; Miller & Stanford, 1987; Sung, 2007). The sex-typing interpretation fits with the interest development model (Gottfredson, 1981; Roe & Siegelman, 1964). In their personality theory of career choice, Roe and Siegelman (1964) postulated that, typically, girls will be more likely to quickly adopt social interest (social scale) and that boys will focus more on interests involving things (realistic and investigative scale). In her theory of circumscription and compromise, Gottfredson (1981, 2002) claimed that sex typing is a primary filter to evaluate experience. Tracey (2002a) found a gender trend: over time, boys became less interested (and perceived themselves as less skilled) in artistic and social areas while girls perceived themselves as less interested in investigative activities. The decrease in math and science interests among girls has been well documented (Catsambis, 1995; Eccles, 1987, 1994; Hilton & Berglund, 1974; Jones, Mullis, Raizen, Weiss, & Weston, 1992; Weinburgh, 1995). Moreover, according to Eccles (1994), boys and girls with similar personality traits may develop divergent occupational interests because of social influences on gender roles (e.g., from parents, teachers, culture, and societal norms). Simply observing that an occupation is performed exclusively by workers of the other sex may well reduce individuals’ likelihood of developing or expressing an interest in that field; this observation suggests the existence of a self-perpetuating cycle that makes it difficult to significantly increase the number of gender-nontraditional workers within fields (Weisgram, Bigler, & Liben, 2010). Other researchers (e.g., Woods & Hampson, 2010) have posited that gender and personality traits jointly influence the development of vocational interests. Identification with parents (Helwig, 1998) and with teachers (Wall, Covell, & MacIntyre, 1999) are also important factors. Parents and teachers shape children’s interests by controlling the type of activities that they are exposed to; through their interactions, teachers and parents influence children’s perceptions of appropriate careers (Bandura et al., 2001). According to Boerchi (2011), it is difficult to determine if these differences are due to social roles, which imposes the development of gender-specific interests, or evolutionary psychology, which maintains that differences are biological and created by sexual selection over time. It is difficult to determine if the differences assessed by interest inventories are real or if they represent socially acceptable expressions of interests which are less gender-determined.

## Assessment of Interests in Childhood and Adolescence

The classic questionnaires are not always suitable for childhood; we can find few guidelines for the assessment of interests with this target.

### Use of Familiar Activities

The literature on children’s interests focuses almost exclusively on expressed aspirations, wherein children are asked what career they would like to have as an adult (Barak, Feldman, & Noy, 1991; Phipps, 1995; Schulenberg, Goldstein, & Vondracek, 1991; Trice, 1991; Trice & King, 1991; Trice & Rush, 1995; Tyler, 1955). Children tend to have sex-typed occupational aspirations (e.g. Tyler, 1955), but there has been little focus on the broader representations of interests. Therefore, the better method to assess interests appears to be a focus on familiar activities, rather than unfamiliar adult occupations. It is better to construct an instrument that includes activities relevant to children’s lives—activities which they engage in and have the necessary experience to accurately assess. For research in which interests have been assessed through familiar activities, the structure of interests in children has been found to differ from that of adults (Dusek, Kermis, & Monge, 1979; Freeberg & Rock, 1973; Tyler, 1955, 1956; Zbaracki, Clark, & Wolins, 1985).

### Possible Use of Pictures

Most interest inventories are based on complex verbal stimuli and call for the examinee to have a considerable degree of verbal proficiency. Pictures, however, are closer to real life than printed statements are. Thus, during testing, pictures should elicit preference responses that are closer to real-life responses. It appears that, in principle, it is possible to construct picture items which are much less ambiguous than mostly verbal items. This decreased ambiguity should lead to higher reliability and improved validity (Geist & McDaniel, 1952).

In the literature, two older inventories used pictures to assess childhood interests: the Geist Picture Interest Inventory (Geist, 1959) and the Reading-Free Vocational Interest Inventory (Becker, 1967).

Some pictorial interest inventories have been created more recently, both for children and adults. These include the PICS and PICS-2 (Picture Interest Career Survey; Brady, 2007); the Career Interest Card Sort (Athanasou & Hosking, 1998), which is a procedure developed for use with adults in employment counselling and vocational guidance; and the Picture Interest Inventory (Stoll, Jungo, & Toggweiler, 2006; Toggweiler, Jungo, & Stoll, 2004), a nonverbal test that uses photographs of people performing vocational activities. The latter inventory, which is mainly used with Swiss adolescents, allows the assessment of vocational interests in terms of Holland’s theory. The Test of Photos of Professions (or Berufsbilder Test [BBT]), a projective method to clarify professional inclination, is used with adolescents (Melo-Silva, Pasian, Assoni, & Bonfim, 2008); a pictorial version of the RIASEC scales of the Personal Globe Inventory (Enke, 2009) is used for adults; and the Pictorial and Descriptive Interest Inventory (PDII; Šverko, Babarović, & Međugorac, 2014) was developed in order to provide students who are finishing elementary school with an Internet-based measure of RIASEC interests.

Finally, in the Italian context, Viglietti produced the MV-70 (Viglietti, 1974), an inventory that is limited by its gender bias; each sector is composed of ten jobs exclusively for males and ten jobs that can also be carried out by women; masculine jobs are more prestigious, and feminine jobs are congruent with gender prejudices.

## Method: Drafting of the Inventory

According to Howard and Walsh (2010), starting from the third level (external activities), children choosing jobs consider interests even if those interests are based on external, observable activities. That is why we have to study and improve conceptualisation on interests, starting with 8-year-old children to promote a linear development of interest conceptualisation which can help students make choices in the future. When this process is not adequate, counsellors meet with those students who are unable to reflect on their interests or understand the differences between training courses when they have to make their first school choice.

That is why we decided to propose a new instrument appositely made for research on children’s interest development; our instrument could be brief, specific, and characterised by simplicity and consistency with childhood targets and the current sociocultural context. The result is a 65-item picture inventory. It takes only a few minutes to be administered and is able to attract the interest of children without tiring them.

The result, the Iconographic Inventory on Professional Interests (3IP), is a self-assessment tool that predominantly uses pictures as stimuli and that is well-suited to be used with younger students and foreign students, both of which are usually characterised by limited language skills.

The process to develop the inventory required three phases. The first was dedicated to the choice of professions to insert in the inventory, the second was a pilot study to define the definitive version of the inventory, and the third was the study to test the psychometric properties of the definitive instrument and to standardise it. Here, they will be briefly described.

### Choosing the Professions

In order to ensure good content validity, the initial phase consisted of a set of professions that, for the target of students ranging in age from 9 to 13, was as much as possible representative of different areas of professional interest. Since we could not refer to a unique, shared model for the areas of interest, we chose to use the PSP/3 – Preferenze Scolastiche e Professionali (Scholastic and Professional Preferences) model (Bonelli & Mancinelli, 2012), an Italian inventory of educational and career preferences that is mostly used in guidance projects to direct 13-year-old students in choosing subsequent studies. A list of 21 professional interest areas was presented to 30 students at primary and secondary schools; the descriptions did not refer directly to any profession. The students were then asked to indicate all the professions.

A list of 75 professions was selected on the basis of the frequency they were mentioned in the PSP/3 model, the students’ knowledge levels, and the consistency that the professions were identified with at least one area of the model. Three more professions were added to cover military areas; these were not part of the PSP/3 model but were fairly frequently cited by students.

### Pilot Study

**Figure 1:**
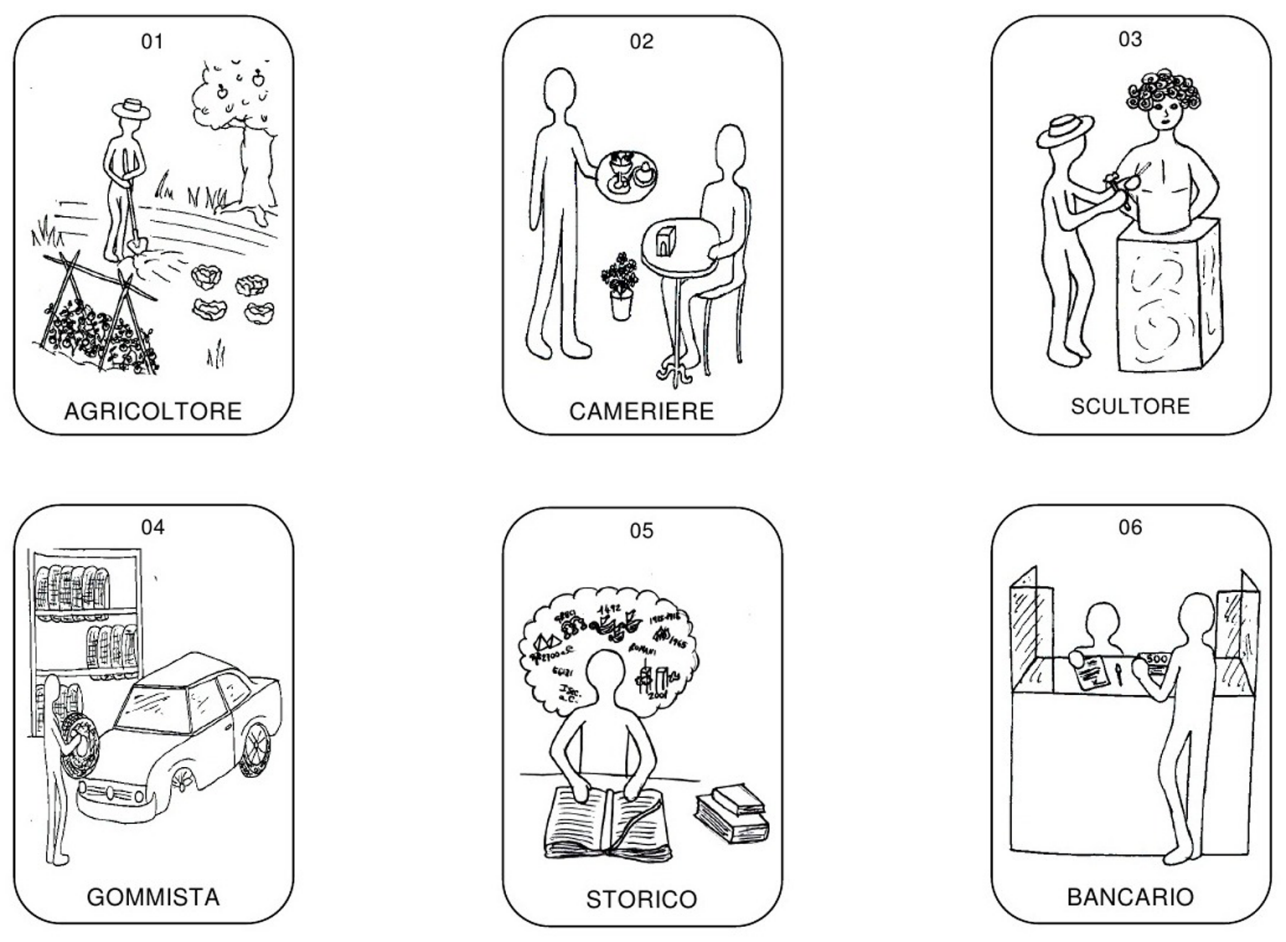
Examples of items.

For each career, a picture was created to represent a person performing that profession. In order to minimise the influence of stereotypes, the human figures in the pictures were stylised, rendering them unidentifiable in terms of gender and facial expression (see examples in Figure 1).

Pictures were accompanied by the name of the profession and a sequential number, and they were collected in a questionnaire. The front page of the questionnaire listed the instrument’s title and instructions for filling it out. The questionnaire was accompanied by a response sheet that was adapted to measure the interest in each profession using a 4-point Likert scale ranging from 1 (*none*) to 4 (*a lot*).

The questionnaire in the pilot study was administered to 143 students. Of these students, 55 were from primary school, and 88 were from secondary school. The participants were equilibrated by gender; 56 were from the north of Italy, and 87 were from the south of Italy.

Assessing interest inventories, data are typically distributed in a nonnormal way with a positive asymmetry. Thus, we tested the distribution obtained by transforming the data with a logarithmic function. Usually, skewness and kurtosis between –1.5 and 1.5 are preferred (Tabachnick & Fidell, 2001), but they are considered acceptable between –2 and 2 (George & Mallery, 2010). The result was a very good normal distribution for 10 items (both skewness and kurtosis lower than 1), an acceptable normal distribution for 65 items (skewness or kurtosis greater than 1 but less than 1.5), and a kurtosis near 2 for three items. Thus, we decided to use the robust unweighted last squares (ULS) method for factorial analysis, and we logarithmically transformed data only for internal consistency and reliability.

Both exploratory factor analysis with ULS and item analysis based on internal consistency (Cronbach’s α) suggested that we should eliminate 10 professions because of their inconsistency with the hypothesised scale and to refer to a 19-vocational-area model. Of the 19 areas, 17 possessed good internal consistency (Cronbach’s α equal or bigger than .70). The remaining two scales had limited internal consistency due to being composed of only two items.

### Definitive Questionnaire

The final version, composed of 65 professions collected in 19 dimensions (Table 1), was administered to a sample of 1,117 students as follows:

- 51.0% males and 49.0% females;
- 30.9% attending primary school and 69.1% attending the secondary school;
- 89.3% aged 9 to 13 years, with a mean age equal to 11.65 and a standard deviation (*SD*) equal to 1.61; and
- 31.4% from the north and 68.6% from the south of Italy.

**Table 1:** Areas and Professions

Area	Professions
**Agriculture**	Farmer; Breeder; Florist; Gardener
**Hoteliers**	Waiter; Barman; Cook; Receptionist
**Arts**	Sculptor; Designer; Painter; Graphic designer; Ceramist
**Automotive**	Tyre repairer; Coachbuilder; Mechanic
**Literature**	Historical; Geographer; Archaeologist
**Economy**	Bank clerk; Business consultant
**Building**	Architect; Surveyor
**Aesthetics**	Hairdresser; Model; Stylist; Tailor; Cosmetician
**Forensic**	Lawyer; Judge
**Computer**	Computer programmer; Computer technician
**Linguistic**	Writer; Journalist; Interpreter; Translator
**Military**	Policeman; Sheriff; Military; Airplane pilot
**Music**	Singer; Musician; Composer; Orchestra conductor
**Health**	Physician; Ophthalmologist; Otorhinolaryngologist; Dentist; Nurse
**Science**	Scientist; Chemical; Biologist
**Social**	Educator; Social Worker; Psychologist
**Technology**	Carpenter; Smith; Repair Technician; Electrician
**Transport**	Trucker; Driver; Taxi Driver
**Tourism**	Tourist Guide; Travel Agent; Hostess

On this occasion, data were also transformed logarithmically to achieve a normal distribution. The result was a very good normal distribution for 21 items (both skewness and kurtosis between –1 and 1) and an acceptable normal distribution for 43 items (skewness or kurtosis between –1.5 and –1.5); only Item 48 (otorhinolaryngologist) had a nonnormal distribution, with skewness equal to 2.0. This item was not considered in the statistical analysis.

Areas were obtained by calculating the means for the original versions of the items in each area. The distribution was normal for 15 scales (with skewness or kurtosis between –1 and 1), and acceptable (between –1.5 and 1.5) for the other four.

**Reliability.** Reliability was estimated by the internal consistency index (Cronbach’s α). As shown in Table 2, in the total sample, most of the indexes were good. Only *economy*, *building*, and *social* were critical.

**Table 2:** Internal Consistency: Cronbach’s α Index

Area	N° items	Original			Sperman-Brown corrected (4 items’ scale)		
		Total	Primary (*n* = 345)	Secondary (*n* = 772)	Total	Primary (*n* = 345)	Secondary (*n* = 772)
**Agriculture**	**4**	.73	.70	.79	.73	.70	.79
**Hoteliers**	**4**	.70	.70	.75	.70	.70	.75
**Arts**	**5**	.80	.78	.83	.76	.74	.80
**Automotive**	**3**	.85	.83	.87	.88	.87	.90
**Literature**	**3**	.73	.76	.76	.78	.81	.81
**Economy**	**2**	**.58**	**.54**	**.62**	.73	.70	.77
**Building**	**2**	**.67**	**.62**	.73	.80	.77	.84
**Aesthetics**	**5**	.87	.88	.87	.84	.85	.84
**Forensic**	**2**	.71	**.65**	.74	.83	.79	.85
**Computer**	**2**	.70	**.66**	.75	.82	.80	.86
**Linguistic**	**4**	.70	.71	.73	.70	.71	.73
**Military**	**4**	.80	**.59**	.82	.80	**.59**	.82
**Music**	**4**	.78	.78	.81	.78	.78	.81
**Health**	**5**	.75	**.69**	.82	.71	**.64**	.78
**Science**	**3**	.79	.80	.84	.83	.84	.88
**Social**	**3**	**.59**	**.53**	.70	**.66**	**.60**	.76
**Technology**	**4**	.80	.74	.86	.80	.74	.86
**Transport**	**3**	.79	.73	.85	.83	.78	.88
**Tourism**	**3**	.70	.70	.75	.76	.76	.80

*Note.* Boldface indicates indexes lower than .70.

If we compare the subsamples of primary and secondary students separately, we obtain a better situation for secondary students (who were critical only for *economy*) but a worse situation for primary students (who were critical for *economy*, *building*, *forensics*, *computers*, *military*, *health*, and *social*). Because it is established that the internal consistency of a scale is influenced by its number of items, and considering that some scales were composed by only two or three items, we corrected the indexes using the Sperman-Brown formula, which assumes that all the scales are composed of four items. The results showed a very good situation for the secondary sample, a good situation for the whole sample (which was critical only in the *social* area), and an acceptable situation for the primary-student sample (which was critical in *military*, *health*, and *social*).

**Discriminant validity: gender differences**. As mentioned above, professional interest is one of the psychological dimensions in which there are the larger gender differences (Boerchi, 2011). We expected to find a statistically significant difference in most of the areas we assessed. Table 3 shows the differences, their significance as measured with the T-Student test, and the effect size measured with Cohen's d.

**Table 3:** Gender Mean Differences and Effect Sizes

Area	Male (*n* = 570)		Female (*n* = 547)				
	*M*	*SD*	*M*	*SD*	Cohen's *d*	Difference	*p*
**Automotive**	1.99	0.91	1.15	0.39	1.31	0.85	.000
**Technology**	1.92	0.76	1.23	0.40	1.20	0.69	.000
**Military**	2.45	0.91	1.65	0.73	0.97	0.80	.000
**Transport**	1.76	0.81	1.19	0.39	0.95	0.57	.000
**Computer**	2.39	0.99	1.77	0.82	0.69	0.62	.000
**Science**	2.13	0.97	1.83	0.83	0.33	0.30	.000
**Literature**	1.96	0.85	1.69	0.75	0.33	0.26	.000
**Buildings**	2.21	0.92	1.93	0.92	0.31	0.29	.000
**Agriculture**	1.74	0.73	1.60	0.62	0.21	0.14	.000
**Economy**	2.05	0.86	1.91	0.82	0.16	0.13	.008
**Forensic**	2.08	0.93	2.00	0.91	0.09	0.08	.127
**Health**	1.74	0.65	1.86	0.65	0.19	-0.13	.001
**Hoteliers**	2.11	0.74	2.26	0.73	0.20	-0.15	.001
**Arts**	1.84	0.71	2.13	0.81	0.38	-0.28	.000
**Linguistic**	1.71	0.65	2.00	0.76	0.41	-0.29	.000
**Social**	1.56	0.59	1.86	0.71	0.46	-0.30	.000
**Music**	1.73	0.79	2.15	0.80	0.53	-0.42	.000
**Tourism**	1.79	0.74	2.24	0.85	0.57	-0.45	.000
**Aesthetics**	1.45	0.51	2.74	0.82	1.94	-1.29	.000

Excluding *forensic*, the means’ differences were all clearly consistent and in the direction we expected: *automotive*, *technology*, *military*, *transport* and *computer* were preferred by males and *aesthetics*, *tourism*, *music*, *social* and *linguistic* were preferred by females. The effect sizes were clearly defined, too: three were very large (*d* > 1.00); three were large (0.66 < *d* < 0.99); five were moderate (0.36 < *d* < 0.65); 7 were small (0.11 < *d* < 0.35); and one was near zero.

**Concurrent validity.** To test the concurrent validity of the scales, the 3IP was administered together with the PSP/3 for a part of the sample (174 students attending the third year of the secondary school; 13–14 years old and with gender nearly equally distributed). Table 4 shows only the correlations which explain more than 33% of the variance in the relationship between two variables, than those bigger than .57.

**Table 4:** Concurrent Validity with PSP/3

SPS/3	3IP																		
	Agriculture	Hoteliers	Arts	Automotive	Literature	Economy	Building	Aesthetics	Forensic	Computer	Linguistic	Military	Music	Health	Science	Social	Technology	Transport	Tourism
**Agriculture**	.74																		
**Hoteliers**		.65																	
**Arts**			.66																
**Handicrafts**			.69																
**Mechanic**				.79													.82	.62	
**Literature**																			
**Economy**						.74													
**Building**							.67										.59		
**Aesthetics**								.73											
**Fashion**								.83											
**Computer**										.81									
**Linguistic**											.70								.62
**Music**													.82						
**Health**														.66		.61			
**Science**															.70				
**Social**																.73			
**Electronic**				.63						.70							.71		
**Transport**				.69								.65					.73	.72	
**Tourism**																			.70
**Graphics**										.61									
**Chemical**															.77				

*Note.* Only correlations which explain more than 33% of variance in the relationship between the two variables are reported.

All of them were statistically significant, with a low error rate (*p* < .001), and congruent with the content of the interest areas. Along the diagonal, you can see the correlations we expected: all were consistent except *literature*, which was only .39 (*p* < .001). Among the areas that were not present in PSP/3, *forensics* was not correlated with any variables, *military* was correlated with *transport*, *graphics* was correlated with *computer*, and *chemical* was correlated with *science*. Most of the variables correlated only with one or two of the PSP/3 variables. Exceptions included the technical areas *automotive*, *computer*, and *technology*.

## Method: Testing New Models

As indicated above, the aim of this research is to test two new models on the same data gathered for the definitive questionnaire.

### Four-Factor Model

As a first step, we tried to identify a second-level structure starting from an exploratory factorial analysis, using the 19 areas as items. Considering that all areas except *transport* had a normal distribution, for this step, we decided to use them without a logarithmic transformation. A principal axis extraction and an Oblimin rotation with Kaiser normalisation EFA were conducted. The oblique solution was chosen, assuming a relationship between the dimensions. The Kaiser criterion (eigenvalues > 1) suggested a four-factor solution which explained 52.6% of the variance. As a second step, the *health*, *computer*, and *linguistic* areas were eliminated because they were not saturated more than .40 by any of the factors. Table 5 shows the rotated loadings of all the areas.

**Table 5:** Model Matrix of Explorative Factorial Analysis

Area	Things	Leisure	Culture	People
Automotive	**.870**	-.170	.061	.024
Technology	**.836**	-.079	-.140	-.042
Transport	**.777**	.068	.095	.087
Agriculture	**.491**	.330	-.152	-.271
Military	**.479**	-.122	-.221	.261
Aesthetics	-.255	**.871**	.147	-.007
Hoteliers	.332	**.606**	.163	.128
Tourism	.027	**.587**	.031	.319
Music	-.049	**.541**	-.289	-.013
Arts	.056	**.486**	**-.454**	-.162
Literature	.019	-.033	**-.758**	.069
Science	.001	-.076	**-.752**	.088
Buildings	.134	.031	**-.420**	.218
Forensic	-.032	-.033	-.195	**.615**
Economy	.150	.181	-.018	**.585**
Social	-.027	.360	-.194	.369

*Note.* Loadings higher than .40 are highlighted, and areas are ordered by factor.

The model explains 53.4% of variance and identifies four main factors, labelled as *things*, *leisure*, *culture*, and *people*. All areas seem to be explained mostly by one factor, with the only exceptions being *arts*—which is bifactorial and will be considered in the *leisure* factor—and *social*, which will be considered in the *people* factor, for which it has the higher loading.

To deeply evaluate the validity of the factors, a model with a confirmatory factorial analysis (CFA) using the maximum likelihood method and AMOS rel.22 software was tested for each of the logarithmically transformed items. Goodness-of-fit indexes were examined through the Chi-square test, RMSEA, and CFI. A nonsignificant Chi-square was desired, suggesting that the observed and reproduced covariance matrices did not differ significantly and thus that they demonstrated a good model fit. However, because the Chi-square test is sensitive to sample size, models with a large sample can only be evaluated with RMSEA and CFI (Byrne, 2010). Models with acceptable fit also present RMSEA < .08 and CFI > .90 (Bentler, 1990), but models with optimum fit present RMSEA < .05 and CFI >.95 (Hu & Bentler, 1999).

**Figure 2:**
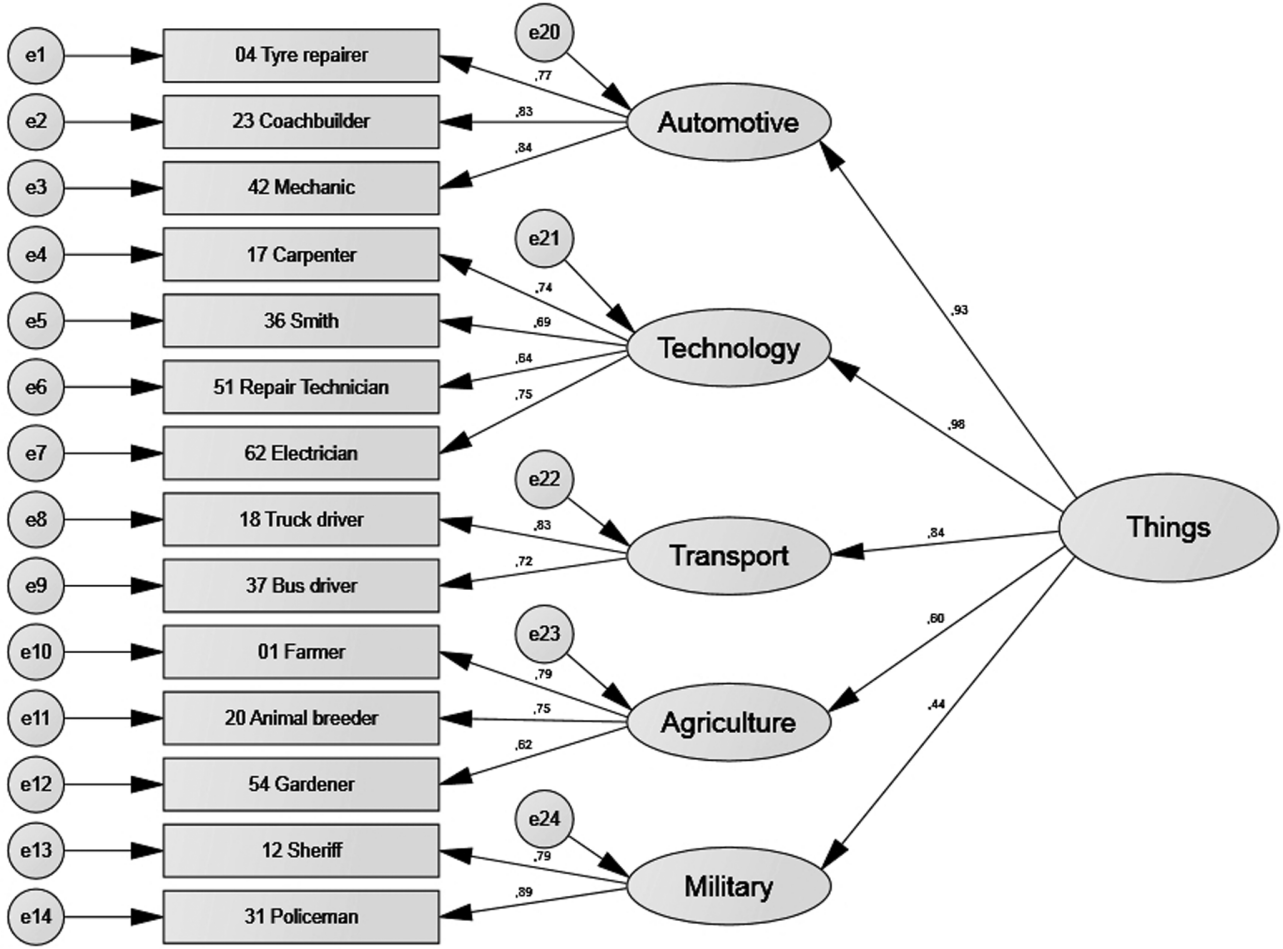
Standardised estimates for the *things* factor.

**Figure 3:**
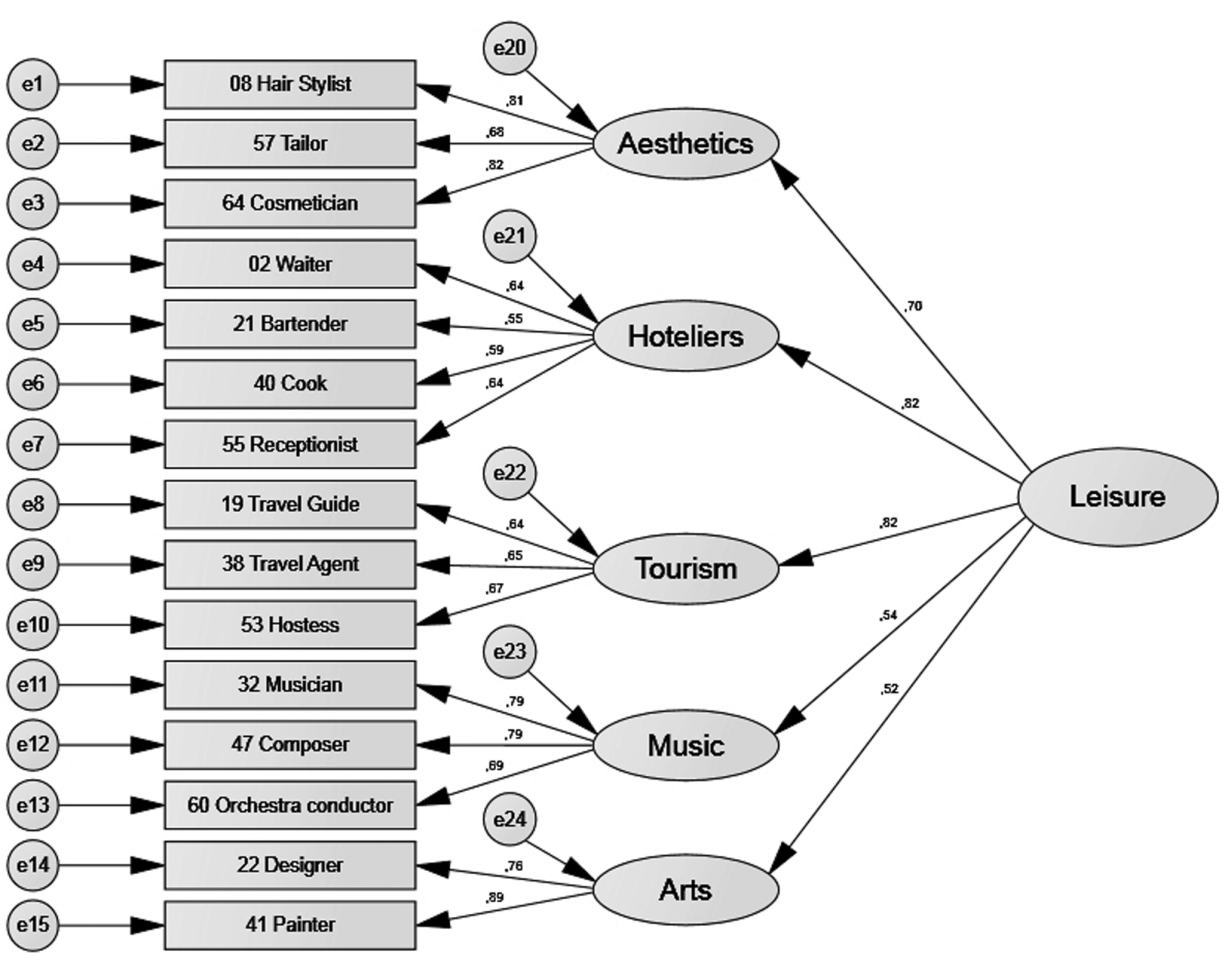
Standardised estimates for the *leisure* factor.

**Figure 4:**
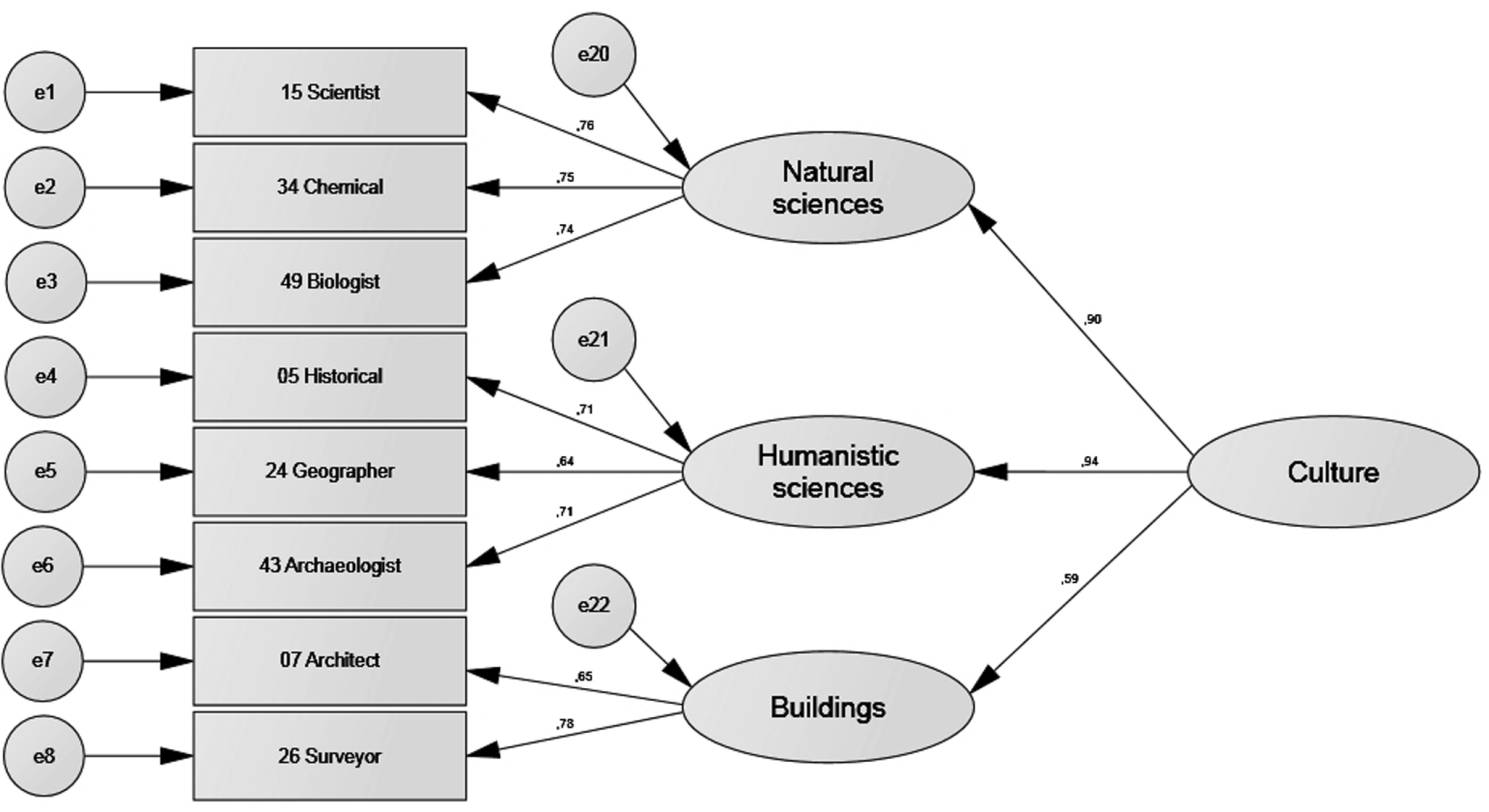
Standardised estimates for the *culture* factor.

**Figure 5:**
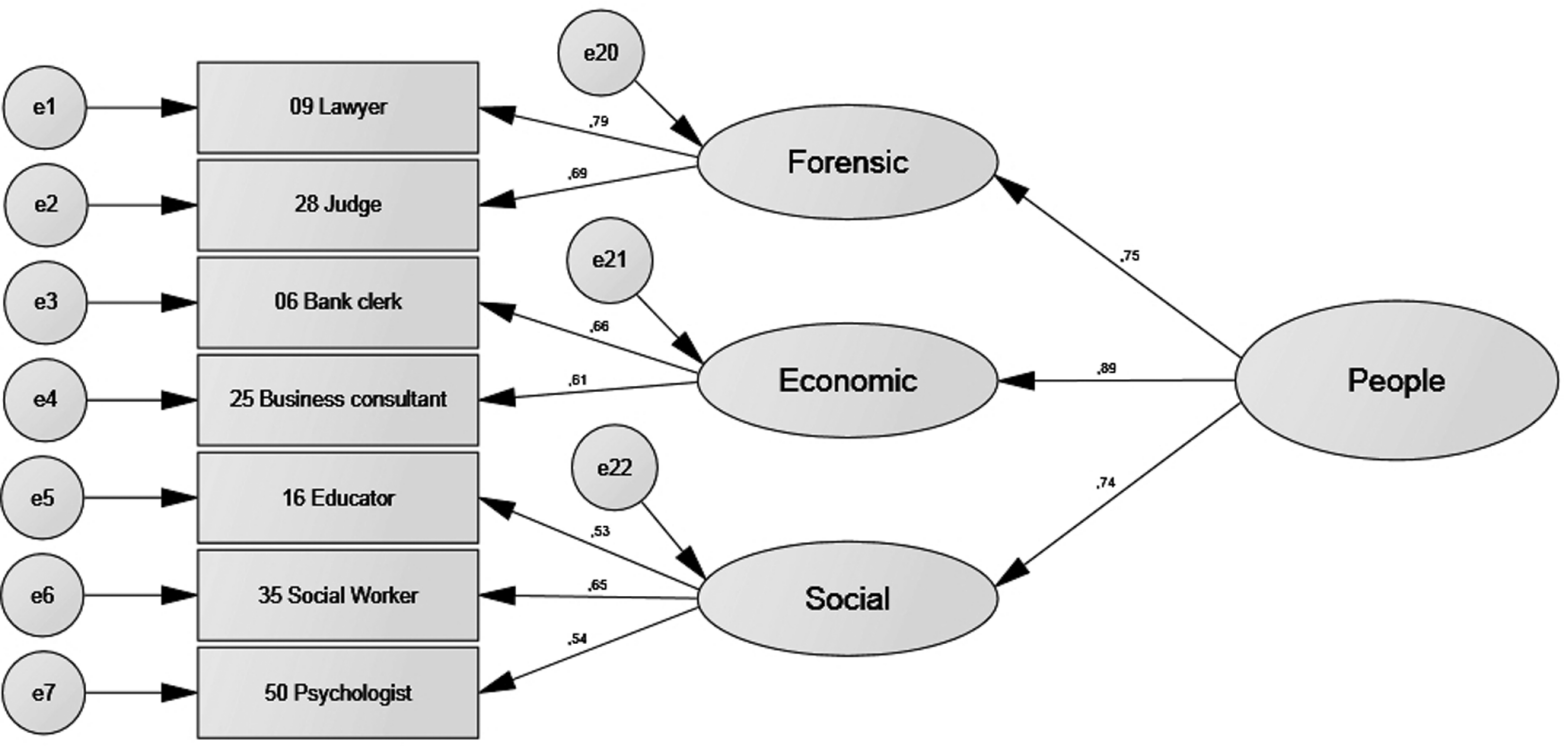
Standardised estimates for the *people* factor.

Table 6 shows that all four models had acceptable or good fit indexes. Figures 2, 3, 4, and 5 show the standardised estimates. To increase the quality of the models, some items with lesser relationships with the latent variable were eliminated. Items 52, 39, 46, and 59 in the *things* factor and Items 27, 44, 13, 3, 56, and 63 in the *leisure* factor were eliminated. No items were eliminated from the *culture* or *people* factors.

**Table 6:** Four-Factor Fit Indexes for CFA Models Tested with Maximum Likelihood (*N* = 1117)

Factor	χ2	*df*	RMSEA	CFI
**Things** (Automotive; Technology; Transport; Agriculture; Military)	402.057***	72	.064 (.058 – .070)	.955
**Leisure** (Aesthetics; Hoteliers; Tourism; Music; Arts)	566.061***	85	.071 (.066 – .077)	.915
**Culture** (Natural sciences; Humanistic sciences; Buildings)	74.553***	17	.055 (.043 – .068)	.980
**People** (Forensic; Economic; Social)	44.110***	11	.052 (.036 – .068)	.977

***p < .001.

### Holland’s RIASEC Model

As for the previous model, as a first step, we tried to identify a second-level structure starting from an exploratory factorial analysis and using the 19 areas as items. A principal axis extraction and Oblimin Rotation with Kaiser normalisation EFA was conducted. The oblique solution was chosen, assuming a relationship between the dimensions. To test the RIASEC model, we forced a six-factor solution which explained 54.2% of the variance. Table 7 shows the rotated loadings of all items. All areas seem to be explained mostly by one factor. Five factors were easy to label: *social*, *realistic*, *investigative*, *artistic*, and *enterprising*. The last factor (*conventional*) can also work, even if it contains jobs that are partly connected to the *artistic* (e.g., architect) and *enterprising* (business consultant) factors.

**Table 7:** Model Matrix of Explorative Factorial Analysis

Area	Social	Realistic	Investigative	Artistic	Conventional	Enterprising
Social	**.699**	-.031	.000	.071	.073	-.063
Health	**.637**	-.055	-.102	.061	.049	.027
Forensic	**.508**	.043	-.028	-.164	-.260	.033
Technology	.012	**-.813**	-.041	.063	-.150	.126
Automotive	-.005	**-.809**	.077	-.080	-.112	.093
Transport	.012	**-.727**	.016	-.110	.005	-.182
Agriculture	-.001	**-.529**	-.063	.317	.168	-.042
Military	.174	**-.447**	-.221	-.183	-.102	-.048
Literature	.017	.002	**-.804**	.031	.005	-.075
Science	.126	-.010	**-.672**	.042	-.060	.055
Arts	.013	-.032	-.141	**.693**	-.211	-.030
Aesthetics	.217	.211	.319	**.513**	.113	-.328
Music	.131	.061	-.121	**.401**	-.009	-.236
Buildings	.050	-.019	-.132	.241	**-.594**	.087
Computer	-.068	-.198	-.147	-.022	**-.467**	-.128
Economy	.284	-.074	.141	-.087	**-.449**	-.232
Tourism	.047	.062	-.081	-.026	-.020	**-.770**
Hoteliers	-.004	-.288	.129	.175	-.034	**-.553**
Linguistic	.274	.162	-.278	.107	-.098	-.360

*Note.* Loadings higher than .40 are highlighted, and areas are ordered by factor.

To evaluate the validity of the factors in depth, two models have been compared with a CFA using the maximum likelihood method. In the first, all areas were inserted directly, with a single score representing the mean of the items which compose it; for the second, logarithmically transformed items were selected to find the best solution (step-by-step, those with lower relations to the latent variable were eliminated until the best solution remained).

**Figure 6:**
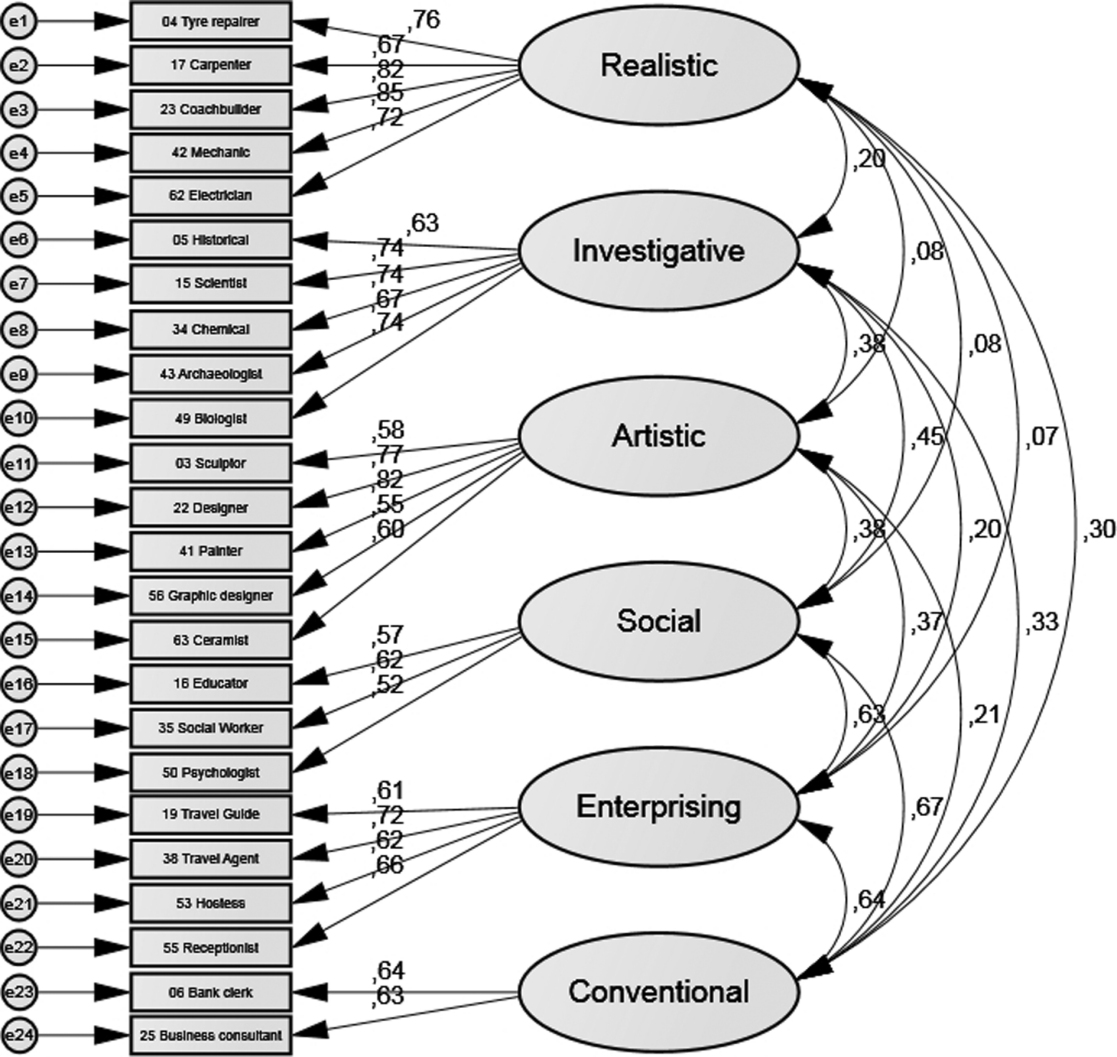
Standardised estimates for the RIASEC model.

Table 8 shows that the first model has bad fit indexes and that the second one, shown in Figure 6, has acceptable fit indexes.

**Table 8:** RIASEC Six-Factor Fit Indexes for CFA Models Tested with Maximum Likelihood (*N* = 1117)

	χ2 (p)	*df*	RMSEA	CFI
**Areas**	2070.655***	137	.112 (.108 – .117)	.757
**Items**	979.845***	237	.053 (.050 – .056)	.920

***p < .001.

Because RIASEC was proposed as a circumplex model, we decided to test this feature, too. The validity of the circular/hexagonal model was investigated using the Hubert and Arabie (1987) randomisation test, performed by RANDALL software (Tracey, 1997). Starting from the correlation matrices between the six factors, the randomisation test (Hubert & Arabie, 1987; Rounds, Tracey, & Hubert, 1992) provided two indices: the correspondence index (CI), a descriptive measure of the model’s degree of fit to the data, and the *p* value, which can be interpreted as the significance of the fit. The CI may vary between 1 and –1; higher CI values imply better fits to the data. Rounds and Tracey (1996) defined the standard for Holland’s model, affirming that the CI must be larger than .66.

For instance, in Šverko and Babarović (2006), after two administrations in 1998 and 2002, the CI values were .72 and .86, respectively, for 18-to-19-year-old students; .57 and .67, respectively, for 16-to-17-year-old students; and .31 for 15-year-old students in 1998. The randomisation *p* values in all samples did not exceed .05. According to the previous literature (Tracey, 2002b; Tracey & Robbins, 2005; Tracey et al., 2006), the authors conclude that “It can be seen that as the students get older, they start to conceptualise interests in ways more like Holland’s model” (Tracey & Ward, 1998, p. 496).

RIASEC variables have been calculated, summing the logarithmically transformed items selected by the second model. All six variables had a good normal distribution; only *realistic* was partly asymmetric (1.255). Starting from the Pearson correlations matrix in Table 9, the CI value was .62 and the *p*-value was .033.

**Table 9:** Pearson Correlations Matrix Between the Six RIASEC Factors

Factor	Realistic	Investigative	Artistic	Social	Enterprising	Conventional
**Realistic**	**.874 (.874)**					
**Investigative**	.188**	**.830 (.830)**				
**Artistic**	.102**	.325**	**.795 (.795)**			
**Social**	.023	.276**	.240**	**.587 (.703)**		
**Enterprising**	.030	.130**	.281**	.372**	**.747 (.787)**	
**Conventional**	.388**	.325**	.172**	.176**	.182**	**.576 (.773)**

*Note.* In the diagonal Cronbach alpha. In brackets indexes corrected with Sperman-Brown prophecy formula assuming all the scales were composed by 5 items.

**p < .01.

## Discussion

3IP is a vocational interest inventory created with a particular attention to content validity. The jobs inserted in the questionnaire were selected to be well-known by students 9 years old and up. Thus, the definitive number of jobs was limited, but the inventory still measures students’ vocational interests in 19 professional areas. A by-product of this is that most of the areas contain a very small number of jobs, with evident consequences on the reliability and validity of the resulting measures. Despite this limit, this research has shown good qualities and the potential to be a powerful psychological inventory to assess second-level areas. Both the four-factor and RIASEC models have good psychometric features. This is an important result considering that the models were not theorised from the beginning. RIASEC, in particular, has shown good fit on the circumplex model: its CI of .62 is not optimal, considering the limit of .66 proposed by Rounds and Tracey (1996), but, considering that our sample was from 9 to 13 years old, this CI reached the level of the Croatian sample Šverko and Babarović (2006) attained at ages 16 to 17. Because this result is partially in opposition to the previous research (e.g., Tracey & Ward, 1998), the question is how much this CI depends on the developmental level and how much it depends on the assessing instrument’s features.

The potential to become a more precise instrument for psychological assessment of vocational interests for younger students can be achieved through a targeted increment of the number of jobs. Increasing each area to five items will likely be enough to obtain good internal reliability, even though this is not enough to guarantee good validity for second-level models. The RIASEC model needs particular attention; new jobs have to be chosen according to the limited number of jobs in some of the factors. The four-factor model could change in terms of both number and content because it probably is not completely representative of all the different kind of jobs a student can be interested in.

## Conclusion

3IP has been shown to possess good psychometric qualities despite the limited number of items in some of its scales. Increasing the number of jobs could both improve the reliability of some scales and enhance the overall capability to assess second-level areas, as described by the RIASEC model. The challenge is to improve the quality of the instrument while maintaining its capacity to work with 9-to-10-year-old students. Thus, particular attention must be paid to the selection of items that are well-known by younger students.
